# A Ruptured Spetzler and Martin Grade V Arteriovenous Malformation in a Child Treated With Radiotherapy Followed by Embolization: A Case Report and Literature Review

**DOI:** 10.7759/cureus.16605

**Published:** 2021-07-24

**Authors:** Kensho Iwatate, Yasuhiro Kikuchi, Sonomi Sato, Mudathir Bakhit, Akio Hyodo

**Affiliations:** 1 Neurosurgery, Fukushima Medical University, Fukushima, JPN; 2 Neurosurgery, Southern Tohoku Research Institute for Neuroscience, Southern Tohoku General Hospital, Koriyama, JPN; 3 Neurosurgery, Dokkyo Medical University Saitama Medical Center, Koshigaya, JPN

**Keywords:** giant avm, hypofractionated stereotactic radiotherapy, postradiosurgical embolization, spetzler-martin grade v, onyx, ruptured pediatric high-grade avms, ruptured arteriovenous malformation, cyberknife, embolization

## Abstract

Treatment of ruptured high-grade Spetzler-Martin (S&M) arteriovenous malformation (AVM) is challenging and requires a multidisciplinary treatment approach. Here, we report a case of ruptured giant callosal Grade V AVM in a child initially treated with stereotactic radiotherapy followed by endovascular embolization with Onyx; a management approach recently described in a few reports on the “postradiosurgical embolization” method. Complete obliteration was achieved 20 months after stereotactic radiotherapy and embolization. In this article, we discuss the usefulness and significance of postradiosurgical embolization, particularly for high-grade AVMs. To our knowledge, this is the first case with a giant Spetzler-Martin Grade V AVM treated with a postradiosurgical embolization method.

## Introduction

The treatment of ruptured pediatric high-grade AVMs is challenging, and until now, there are no agreed-upon protocols for its management. A multidisciplinary approach with the combination of radiotherapy, embolization, excision, and clinical follow-up is commonly practiced [1,2]. In high-grade AVMs, the larger the size, the lower the occlusion rate, and the more it becomes difficult to treat [3]. Volume-reductive embolization followed by radiotherapy, so-called preradiosurgical embolization, is the standard treatment method for high-grade AVMs unsuitable for surgery. While the efficacy of preradiosurgical embolization has been reported in several studies [4,5], other studies have described lower obliteration rates with this technique compared to the radiation-only method [6,7]. Various factors of lower obliteration rates after preradiosurgical embolization have been reported, including incomplete targeting of the nidus due to embolic material artifacts [4], energy attenuation caused by embolic material [5,8], and recanalization of non-irradiated embolic sites [9].

Recently, a few reports suggested a different approach to manage AVMs, the postradiosurgical embolization method, where embolization is performed after radiation therapy. This approach has been developed to overcome the disadvantages of preradiosurgical embolization [10]. Here, for the first time, we report a successful complete occlusion of ruptured giant callosal AVM in a child treated with stereotactic radiotherapy followed by postradiosurgical embolization.

## Case presentation

Clinical presentation and diagnosis

**Figure 1:**
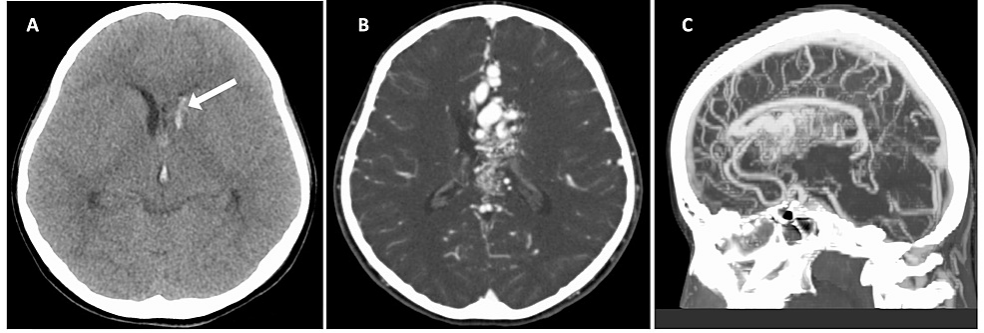
Preoperative CT (A) Preoperative CT shows the intraventricular hemorrhage centered in the left anterior horn (arrow). (B) Preoperative contrast-enhanced CT axial image demonstrating a 7.5 cm length AVM spreading in the cavum septum pellucidum, corpus callosum, and left ventricular body. (C) Preoperative contrast-enhanced CT sagittal image showing high flow feeder from anterior cerebral artery and deep venous drainage to the inferior sagittal sinus.

A 12-year-old girl presented with headaches and mild disorientation [Glasgow Coma Scale (GCS): E3V5M6]. A plain brain CT scan showed an intraventricular hemorrhage (Figure 1A), and the contrast-enhanced CT confirmed a large AVM mainly in the corpus callosum (Figures 1B-1C).

**Figure 2:**
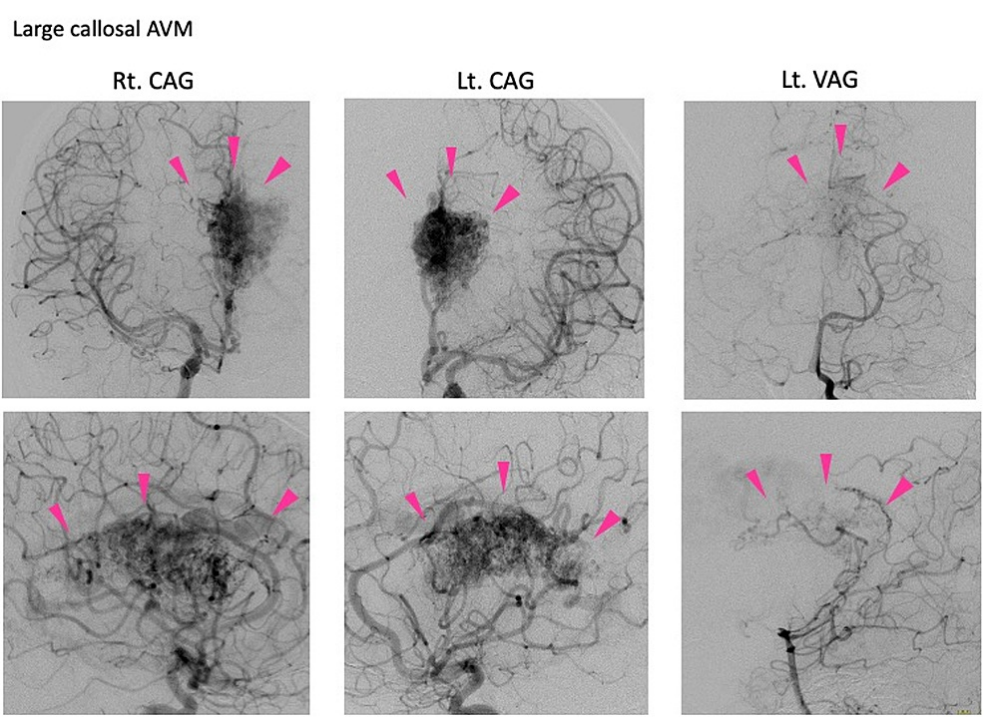
Preoperative angiography Right cerebral angiography (Rt. CAG), left cerebral angiography (Lt. CAG), and left vertebral angiography (Lt. VAG) show a 7.5 cm diameter AVM. The pink triangle points to the AVM's nidus. The feeder is from Lt. ACA with a high flow.

Cerebral angiography showed that the main feeder was from the left anterior cerebral artery (Lt. ACA). Furthermore, flow came from the right anterior cerebral artery (Rt. ACA) and left posterior cerebral artery (Lt. PCA). The drainer was deep, mainly towards the inferior sagittal sinus and basal vein of Rosenthal. The AVM’s maximum diameter was 7.5 cm, and its Spetzler-Martin (S&M) grade was V (large size, eloquent area, deep venous drainage) (Figure 2).

Because of the AVM location, which is widely extended to a large area of the corpus callosum, surgical excision was expected to cause a significant neurological deficit for the patient. Furthermore, with a nidus volume up to 17.3 ml, achieving a complete cure by radiotherapy alone was expected to be challenging. Therefore, we decided to treat the patient with CyberKnife M6 ((Accuray, Sunnyvale, CA, USA) radiotherapy followed by Onyx embolization, the so-called postradiosurgical embolization method.

Treatment

**Figure 3:**
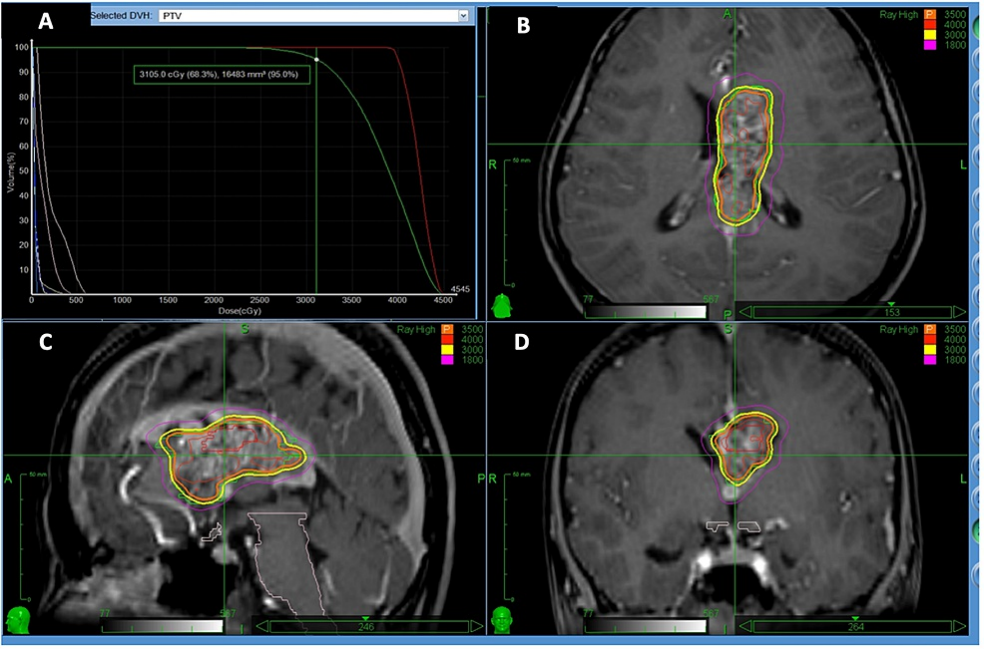
Dose-planning of stereotactic radiotherapy Through CyberKnife M6, the AVM with a GTV of 17.3 ml received 35 Gy in five fractions, with an isodose line of 77%. Planned target volume (orange) covered by the 77% isodose can be seen in axial (B), sagittal (C), and coronal (D) planes on pretreatment planning computed tomography angiography (CTA). The upper left graph (A) shows that 95% of the area was irradiated with more than 31 Gy. The maximum dose was 45 Gy.

After 135 days from the onset of symptoms, stereotactic radiation of the AVM was done with a gross tumor volume (GTV) of 17.3 ml using CyberKnife M6 with 35 Gy in five fractions and an isodose line 77%. In order to reduce the blood flow to the nidus, we applied 35 Gy focusing on the area where the feeder flows into the nidus (Figure 3).

**Figure 4:**
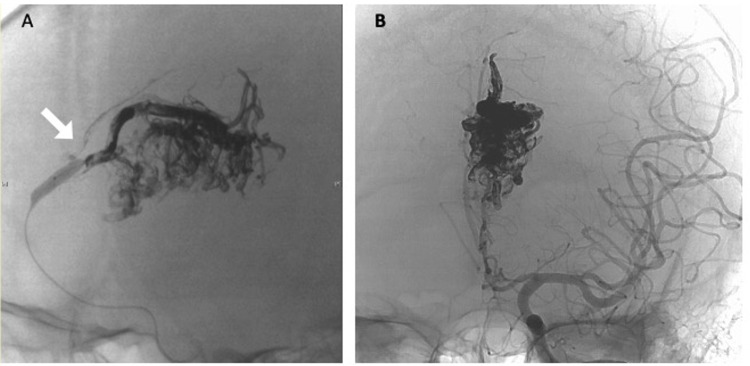
Angiography during embolization (A) Lateral view and (B) anteroposterior view. Onyx18 being injected through a microcatheter (Scepter XC: 4 mm x 11 mm) inserted in the Lt. ACA and refluxed back through the space between the inflated balloon and the blood vessel, causing an accidental occlusion of a branch of Lt. ACA (white arrow).

Fifteen days later, we performed the endovascular embolization with Onyx. We guided the extra-compliant balloon microcatheter (Scepter XC, Microvention, Tustin, CA, USA) into the main feeder, the Lt. ACA, and injected Onyx18 (ev3, Irvine, CA, USA) while the balloon of the microcatheter was inflated (Figure 4). In the early stages of Onyx embolization, the Onyx filled the nidus smoothly. However, in the later stages of embolization, the injection resistance gradually increased. To counter this, we increased the injection pressure. Consequently, some of the Onyx material refluxed through the balloon and the vessel wall gap. Thus, accidentally, a distal branch of Lt. ACA was occluded, and therefore we immediately terminated the procedure at that stage (Figure 4).

**Figure 5:**
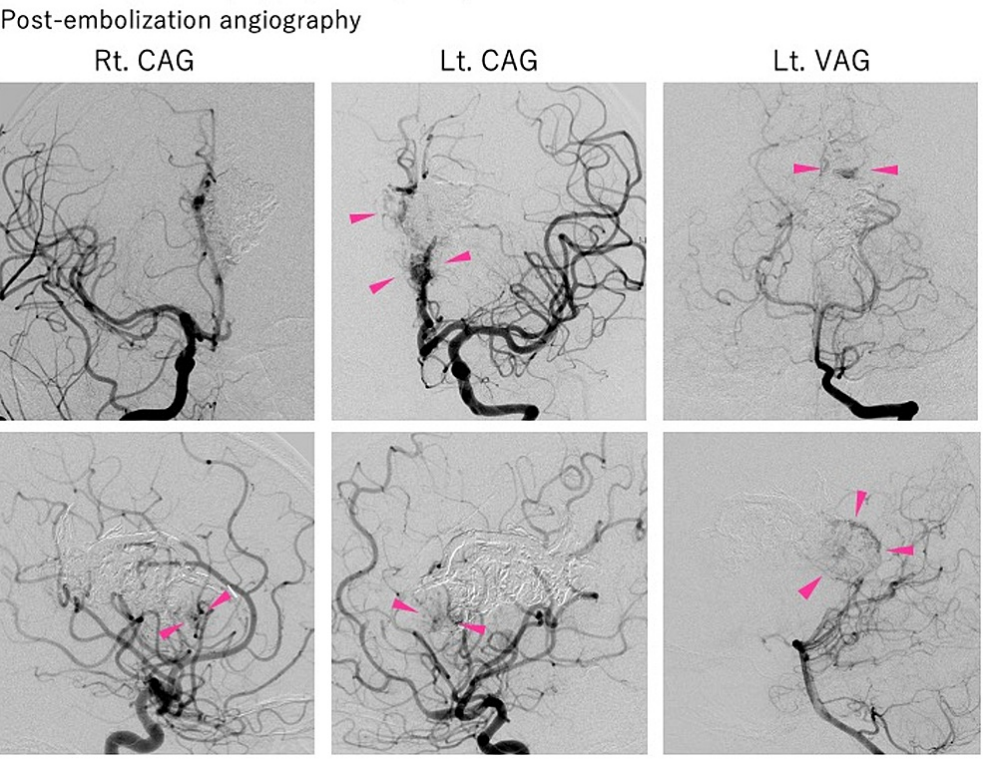
Post-embolization angiography Angiography after Onyx embolization. Rt. CAG and Lt. CAG show residual lesions in the anterior inferior part and the right-center part of the nidus (Pink arrow). Lt. VAG shows residual lesions in the left posterior part of the nidus (Pink arrow). The radiological findings suggesting occlusion of approximately 50% of the AVM nidus.

A total of 9 ml of Onyx18 agent was injected into the AVM, and approximately 50% of the nidus was embolized. The residual lesions of AVM nidus were on the anterior-inferior, right-center, and left posterior (Figure 5).

**Figure 6:**
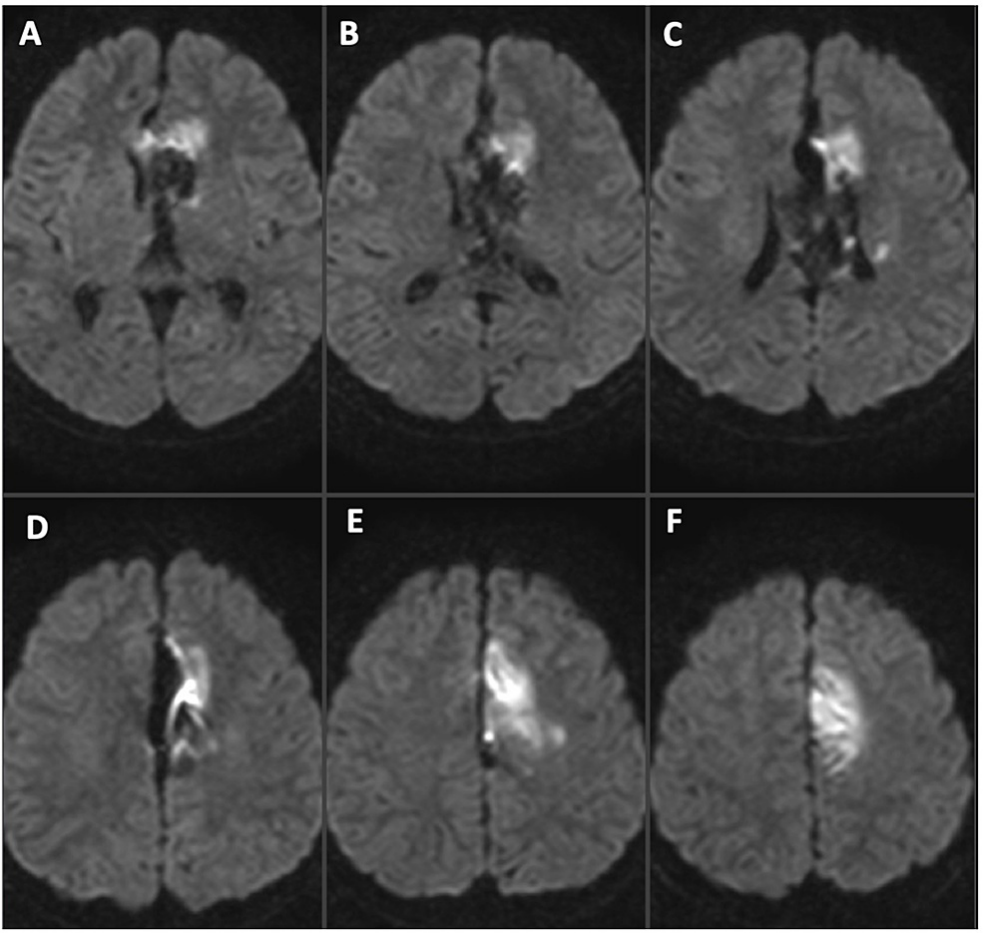
Post-embolization MRI (DWI) Post-embolization diffusion-weighted imaging (DWI) demonstrating infarction of Lt. ACA branch feeding area. Slice is from thalamus (A) level to leg area of the primary motor cortex (F). Infarct area was supplementary motor cortex to primary motor cortex of the leg area (E)(F), cingulate gyrus (D), and corpus callosum (A)(B)(C).

Post-embolization MRI showed partial infarction at the medial part of the left primary motor cortex extending anteriorly to the supplementary motor area. The radiological finding was manifested clinically as a manual muscle testing Grade 3 right lower limb weakness (Figure 6).

**Figure 7:**
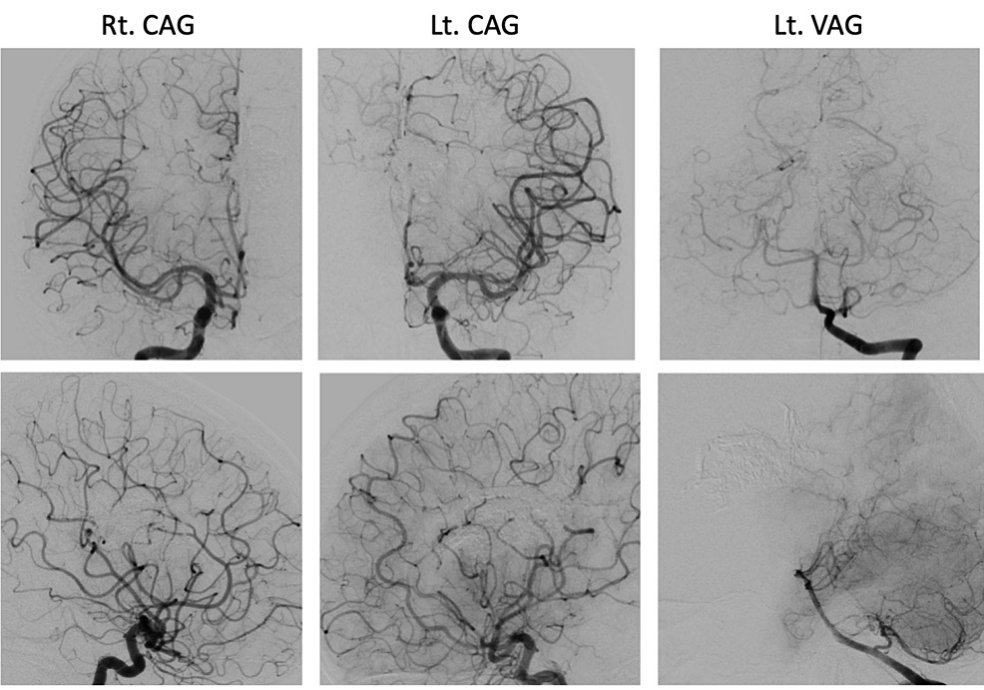
Follow-up angiography after treatment Rt. CAG, Lt. CAG, and Lt. VAG confirmed the complete resolution of the large callosal AVM.

Rehabilitation was performed for the right lower limb weakness, and six months later, the patient was fully recovered. No other neurological deficit was detected. At 20 months after embolization, cerebral angiography showed complete resolution of the large callosal AVM (Figure 7).

Five years after radiation and embolization, no recurrence was seen on follow-up MRI. The left lower extremity weakness has recovered, with no sequelae or new complications.

## Discussion

To our knowledge, this is the first report to describe the management of an S&M Grade V AVM that is completely resolved and managed by radiation therapy followed by endovascular embolization (postradiosurgical embolization).

In a meta-analysis of 3923 cases of cerebral AVMs, a history of hemorrhage, deep brain localization, drainage only into deep veins, and presence of aneurysm were shown statistically predominant risk factors for bleeding [11]. In our case, the following risk factors for rebleed existed: prior hemorrhage, deep AVM location, exclusively deep venous drainage.

Although there is a lack of a guideline, several reports recommend a multimodality approach treatment for large AVM [1,12]. Chang et al. studied 53 large AVM cases with a maximum diameter greater than 6 cm. Most of the patients were treated with combined treatment (embolization followed by surgery in five cases, embolization followed by radiosurgery in 23 cases, and embolization, radiosurgery, and surgery in 23 cases). Nineteen cases had a complete occlusion, four cases had 90% occlusion, 29 cases had less than 90% occlusion, and the treatment-related morbidity rate was 15%. The clinical prognosis at 37 months was excellent in 51%, good in 28%, poor in 6%, and 15% mortality [12]. Other reports described poor outcomes after microsurgery for high-grade AVM was found to be 31% for Grade IV and 37% for Grade V [1,13]. In our case, the maximum diameter of the AVM was 7.5 cm, and it was expanding through the corpus callosum. Accordingly, surgical excision was judged to have a high risk of morbidity. Therefore, we chose the combined radiotherapy and endovascular approach.

Various radiotherapy methods have been reported large and challenging to treat AVM. The commonly used methods are single-stage stereotactic radiosurgery (SS-SRS), volume staged SRS (VS-SRS), and dose staged SRS (DS-SRS). The most common radiotherapy methods for large AVM are VS-SRS and DS-SRS. For VS-SRS, a gamma knife is basically used, and the mean marginal dose varies from 15 to 20 Gy, and the number of stages of irradiations is often reported to be two to four stages [14,15]. For dose staged SRS, a linear accelerator (LINAC) is used, and the mean total dose varies from 26 to 50 Gy, and the number of stages of irradiation varies from two to 12 stages [16,17]. The mean complete occlusion rate was 32.3% for DS-SRS and 41.2% for VS-SRS [15]. However, the same report mentioned that VS-SRS was associated with a more extended treatment period and risk of rebleeding (10.6% with DS-SRS, 19.5% with VS-SRS). In the current case, we chose DS-SRS.

Veznedaroglu et al. compared the outcome of seven AVM cases with a mean nidus volume of 23.8 ml who received 42 Gy in six fractions of 7 Gy with 23 AVM cases with a mean nidus volume of 14.5 ml of nidus who received 30 Gy in six fractions of 5 Gy. The complete occlusion rate obtained after more than five years of embolization was 83% in the 7 Gy group versus 22% in the 5 Gy group. However, complications associated with irradiation were 28% in the 7 Gy group and 4.3% in the 5 Gy group. This study concluded that for large AVM, high conformality is necessary for fewer complications and higher cure rates [16]. A margin dose of 17 Gy or more for volume staged stereotactic radiosurgery more than doubles the partial response rate, reduces the time to complete occlusion by less than half, and increases the probability of complete or nearly complete occlusion [18]. 

The current case’s AVM was 7.5 cm in diameter. Accordingly, we irradiated it with a 35 Gy in five fractions, 77% isodose line, with a minimum marginal dose of 30 Gy. Firlik et al. introduced the method of staged volume radiosurgery for giant AVM, and irradiation was performed from at feeder side to avoid pressure rise in the nidus [19]. The same method was applied in our case. 

Regarding the time to treat an AVM, Maruyama et al. found that there was no benefit in postponing treatment until hematoma resorption because the number of bleeding events was predominantly higher in patients with a median follow-up of six months or longer [20]. In our case, radiotherapy was performed 135 days and endovascular embolization 150 days after bleeding.

Large AVMs are often challenging to treat with embolization alone or radiation alone, and the traditional strategy has been first to reduce the size of the AVM with embolization and then add radiation therapy. This will help to reduce the amount of radiation exposure to surrounding structures. However, many reports show lower obliteration rates in the embolization followed by the radiotherapy group compared to the radiotherapy alone group [6,9]. In general, cases in which embolization is performed before radiation therapy tend to be larger and to have a lower radiation dose per unit volume of AVM than cases in which radiation therapy alone was performed. To overcome the disadvantages of early embolization and the bleeding rate before occlusion after radiotherapy, a strategy of embolization before radiotherapy, i.e., postradiosurgical embolization, might be useful [10]. 

Kim et al. compared 81 patients treated with only Gamma knife radiosurgery (GKRS) with 17 patients treated with GKRS followed by embolization for ruptured AVM. In the ruptured S&M Grade III and IV AVM cases, the rate of occlusion was higher in the group treated with GKRS followed by embolization than in the group treated with GKRS alone [10]. In the current case, although the Onyx embolization could only obliterate about 50% of the Grade V AVM, the nidus gradually shrank due to the effect of the stereotactic radiation, and complete occlusion was achieved and confirmed on angiography 20 months later.

## Conclusions

We present a ruptured giant high-grade callosal AVM in a child cured by postradiosurgical embolization for the first time. Postradiosurgical embolization can be an effective treating strategy for high-grade ruptured AVMs. This method enables accurate radiation planning, early cure, and a low recurrence rate. Further studies of postradiosurgical embolization in larger samples remain necessary to validate the usefulness and applicability of this method. 
